# Flavonoid *O*-Methyltransferases in *Eucalyptus*—Biosynthesis of Alpinetin via a Methylated Chalcone Precursor

**DOI:** 10.3390/ijms27115078

**Published:** 2026-06-04

**Authors:** Liyuan Zhu, Guillermo Garcia-Gimenez, John Humphries, Adam W. E. Stewart, Spencer J. Williams, Jason Q. D. Goodger

**Affiliations:** 1 School of Agriculture, Food and Ecosystem Sciences, University of Melbourne, Parkville, VIC 3010, Australia; liyuan.zhu@student.unimelb.edu.au (L.Z.); guillermo.garciagimenez@unimelb.edu.au (G.G.-G.); 2 La Trobe Institute for Sustainable Agriculture & Food, La Trobe University, Bundoora, VIC 3086, Australia; j.humphries@latrobe.edu.au; 3 School of Chemistry and Bio21 Molecular Science and Biotechnology Institute, University of Melbourne, Parkville, VIC 3010, Australia; asstew@student.unimelb.edu.au (A.W.E.S.); sjwill@unimelb.edu.au (S.J.W.)

**Keywords:** bioactives, cardamomin, cardamonin, enzyme regioselectivity, flavokawain B, flavone, monocalypt, natural products, pinocembrin dimethyl ether, SAM-dependent methyltransferase

## Abstract

Methylated flavonoids are abundant phytochemicals in *Eucalyptus* and are of interest because methylation can alter flavonoid diversity, bioactivity, and stability. However, the enzymes responsible for flavonoid methylation in eucalypts remain largely uncharacterised. We used comparative leaf transcriptomics of two species with contrasting flavanone profiles, together with protein-structure-guided candidate selection, to identify prospective *O*-methyltransferases involved in methylated flavonoid biosynthesis. Five candidate methyltransferases from *E. eugenioides* were cloned, heterologously expressed, and assayed against a panel of flavonoids and a chalcone precursor. The enzymes showed distinct substrate preferences and regioselectivities. *Ee*OMT1 acted as a broad 7-*O*-methyltransferase, whereas *Ee*OMT3–*Ee*OMT5 preferentially methylated B- and C-ring hydroxyl groups, with differing capacities for sequential methylations at different sites. *Ee*OMT2 was of particular interest because it methylated pinocembrin chalcone to alpinetin chalcone more efficiently than it converted the flavanone pinocembrin to alpinetin. Expression–metabolite analyses across *E. eugenioides* genotypes were consistent with roles for *Ee*OMT2 and *Ee*OMT1 in the *in planta* accumulation of 5-*O*- and 7-*O*-methylated flavanones, respectively. These findings support a revised model in which alpinetin biosynthesis proceeds, at least in part, through methylation of a chalcone precursor before flavanone formation. This work provides a foundation for elucidating flavonoid methylation pathways in plants and for engineering the production of tailored methylated flavonoids.

## 1. Introduction

**Figure 1:**
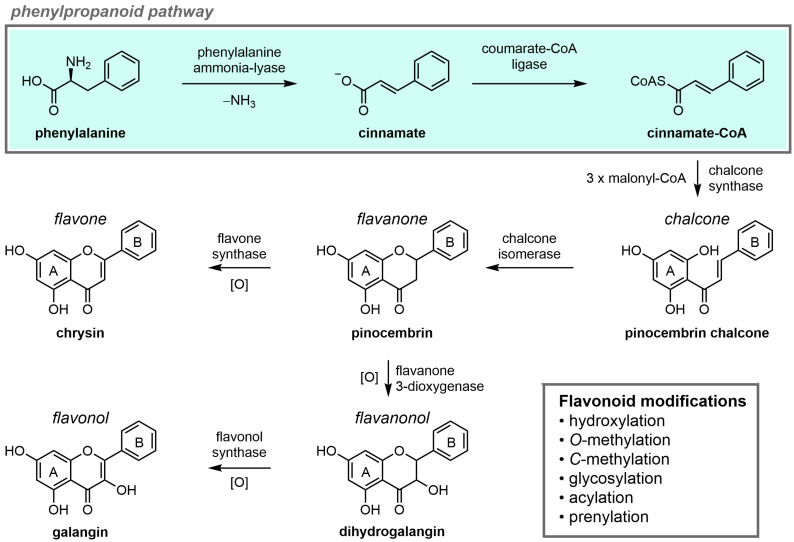
Overview of flavonoid biosynthesis from phenylalanine, showing the phenylpropanoid route to cinnamate-CoA, followed by chalcone formation and conversion to representative flavanones, flavones, flavanonols and flavonols. Common downstream flavonoid modifications include hydroxylation, *O*- and *C*-methylation, glycosylation, acylation and prenylation.

Flavonoids are chemically diverse specialized metabolites with physiological roles in plants that include UV photoprotection and pathogen defence [1,2,3]. Many have antioxidant and anti-inflammatory activities relevant to food, nutraceutical, and pharmaceutical applications [4,5,6,7]. The flavonoid skeleton is produced through the phenylpropanoid biosynthetic pathway, and their structural and functional diversity is expanded by enzymatic tailoring reactions, including hydroxylation, methylation, glycosylation, prenylation, and acylation (Figure 1; [8]). Flavonoid methylation is catalysed by *S*-adenosyl-L-methionine (SAM)-dependent *O*- and/or *C*-methyltransferases (OMTs and CMTs; [9]), and can alter metabolic stability, membrane permeability, and resistance to enzymatic degradation, properties that are relevant to bioactivity and potential applications [10,11,12]. For example, *O*-methylation of flavones has been shown to enhance their membrane-penetrating capabilities, thereby improving their bioavailability as therapeutics [11].

*Eucalyptus* trees (>800 species [13]; Family Myrtaceae) are rich sources of methylated flavonoids, including *O*- and *C*-methylated flavanones and flavones [14,15,16,17]. In monocalypt eucalypts (species of subgenus *Eucalyptus*), methylated flavanones accumulate in leaf surface waxes and in the terpenic oils housed within copious foliar oil glands [18,19]. These profiles are often species- or genotype-specific. For example, some species are incapable of producing methylated flavonoids, others produce only *O*- or *C*-methylated derivatives, some produce derivatives methylated at only one position, whereas others produce mixtures of more than 10 different flavonoids *O*- and *C*-methylated at many different positions [14,15,16]. This chemical diversity makes eucalypts a useful system for linking flavonoid variation with candidate biosynthetic genes, including those encoding plant flavonoid methyltransferases.

Plant OMTs that methylate flavones and flavonols have been relatively well characterised and often show defined regioselectivity patterns [20,21,22,23,24]. By contrast, flavanone OMTs remain comparatively understudied. A flavanone 7-OMT from *E. nitida* (*En*OMT1) was shown to convert the non-methylated flavanone pinocembrin to the 7-*O*-methylated flavanone pinostrobin [25]. No plant enzyme has yet been characterized for formation of the 5-*O*-methylated pinocembrin derivative alpinetin, an important pharmacological component of many traditional Chinese medicines and patent drugs [26]. Although a recent report showed engineered bacterial OMT variants can methylate the 5-hydroxyl of pinocembrin to generate alpinetin, their wild-type counterpart had low activity and low yield (<10%; [27]), suggesting that direct methylation of the flavanone may not be the preferred biosynthetic route in nature.

An alternative possibility is that methylation occurs on a chalcone precursor before flavanone ring closure, where the hydroxyl group corresponding to flavanone 5-OH may be more accessible. Recent work on methylated flavonoids in *Rhododendron dauricum* showed that *C*-methylation in plants can occur on a precursor chalcone, followed by isomerisation into the corresponding methylated flavanone [28]. Thus, the search for an OMT involved in alpinetin biosynthesis should consider pinocembrin chalcone as a potential substrate (Figure 1).

In this study, we used comparative transcriptomics of eucalypts with contrasting flavanone profiles, together with protein modelling and recombinant enzyme assays, to identify five candidate OMTs involved in methylated flavonoid biosynthesis. By testing both flavanone and chalcone substrates, we investigated whether alpinetin formation proceeds through methylation of pinocembrin chalcone before flavanone formation, rather than through direct methylation of pinocembrin. We identify *Ee*OMT2 as a candidate chalcone OMT and our results support a revised route to alpinetin and related 5-*O*-methylated flavanones via methylation of a chalcone precursor.

## 2. Results

### 2.1. Variation in Flavanone Abundance and Diversity Between Eucalypts

**Figure 2:**
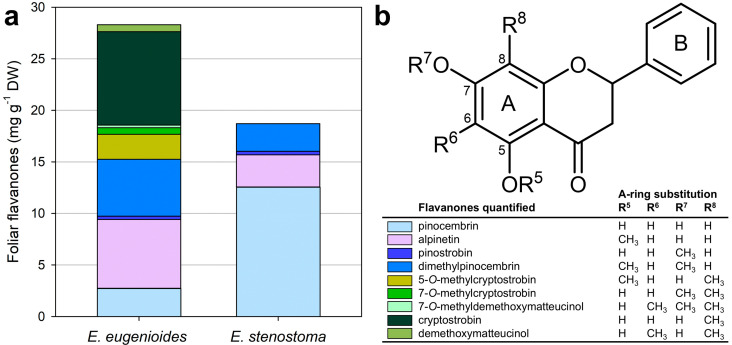
Foliar flavanone profiles of the two eucalypt species selected for comparative transcriptomic analysis. (**a**) Mean total flavanone content and relative flavanone composition in leaves of *E. eugenioides* and *E. stenostoma*. Values are expressed as mg g^−1^ dry weight (DW). Descriptive statistics for the data are presented in Table S1. (**b**) General flavanone scaffold showing the positions of observed *O*-methylation (R^5^, R^7^) and *C*-methylation (R^6^, R^8^) on the A-ring. The R-group table defines the substitutions corresponding to the flavanones detected in the foliar extracts.

Two monocalypt eucalypts, *E. eugenioides* (thin-leaved stringybark) and *E. stenostoma* (Jillaga ash), were selected for analysis based on their contrasting foliar flavanone profiles. On average, *E. eugenioides* accumulated a higher total flavanone content and displayed a more chemically diverse profile, comprising *C*-methylated and 5-*O*-methylated and 7-*O*-methylated flavanones (Figure 2, Table S1). The major components included the *C*-methylated flavanones cryptostrobin (33%) and demethoxymatteucinol (3%), the 5-*O*-methylated flavanones alpinetin (23%) and 5-*O*-methyl cryptostrobin (8%), and smaller amounts of the 7-*O*-methylated flavanones 7-*O*-methylcryptostrobin (3%), pinostrobin (1%), and 7-*O*-methyldemethoxymatteucinol (1%). The *O*-methylated flavanones 5-*O*-methylcryptostrobin and 7-*O*-methylcryptostrobin were isolated and structurally characterised by single crystal X-ray diffraction (Appendix A, Figure S1; Table S9). While 7-*O*-methylcryptostrobin has previously been reported from eucalypts [16], this is, to our knowledge, the first report of 5-*O*-methylcryptostrobin in the genus.

In contrast to *E. eugenioides*, *E. stenostoma* contained lower total flavanone levels and a simpler profile dominated by pinocembrin (67%), with alpinetin and dimethylpinocembrin (5,7-dimethoxyflavanone) also present as significant minor components (16% and 15%, respectively; Figure 2a). These differences, particularly the higher abundance and broader representation of 5-*O*- and 7-*O*-methylated flavanones in *E. eugenioides*, provided a basis for comparative transcriptomic analysis aimed at identifying candidate *O*-methyltransferases involved in flavanone methylation.

### 2.2. Comparative Transcriptomic Analysis

**Figure 3:**
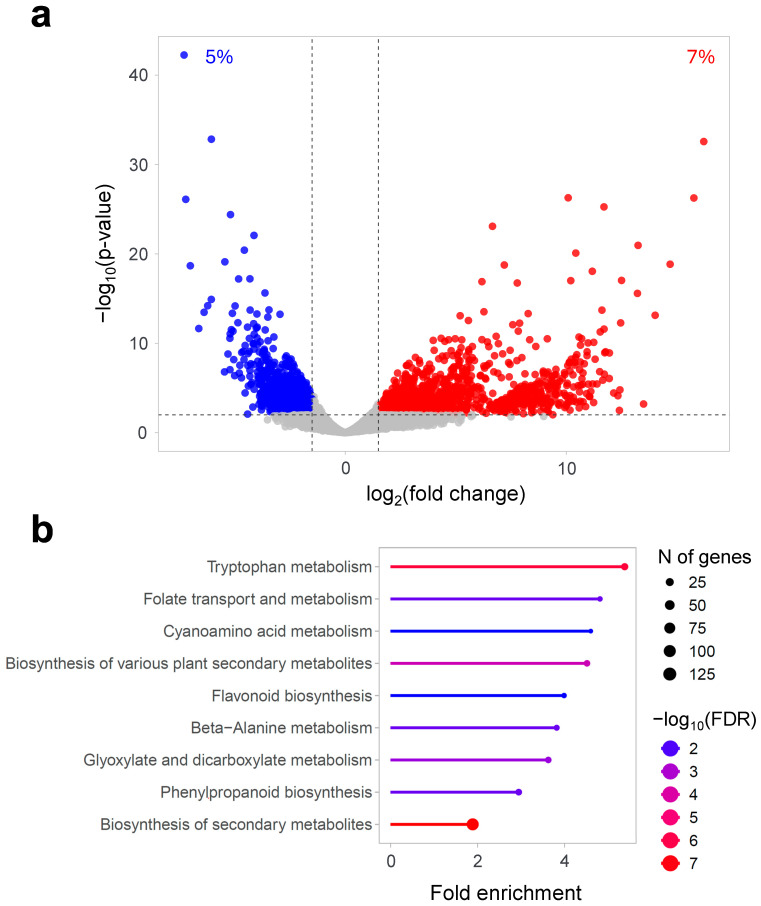
Comparative transcriptomic analysis of eucalypt leaves. (**a**) Volcano plot showing pairwise differential gene expression between *E. eugenioides* and *E. stenostoma*. Red and blue dots indicate genes significantly upregulated in *E. eugenioides* and *E. stenostoma*, respectively; grey dots indicate genes that did not meet the significance thresholds. Dashed lines show the applied cut-offs (*p*-value < 0.01 and absolute log_2_FC > 1.5). The percentage of significantly upregulated genes in each species is shown above the corresponding wing of the volcano plot. (**b**) KEGG analysis of the top 1000 differentially expressed genes (DEGs). The nine most enriched pathways passing the >1.5-FE threshold are shown. Bubble size indicates the number of DEGs assigned to each pathway, FE indicates enrichment relative to the background gene set (*n* = 19,878), and colour indicates statistical significance, expressed as −log_10_FDR.

To investigate foliar transcriptomic differences between *E. eugenioides* and *E. stenostoma*, pairwise differential expression analysis was performed using the *E. grandis* reference genome [29]. This identified more than 19,800 expressed genes. Applying thresholds of *p*-value < 0.01 and absolute log_2_ (fold change) >1.5 revealed 1307 genes significantly upregulated in *E. eugenioides* and 890 genes in *E. stenostoma*, corresponding to 7% and 5% of expressed genes, respectively (Figure 3a). Kyoto Encyclopedia of Genes and Genomes (KEGG) pathway enrichment analysis was then performed using the top 1000 differentially expressed genes to identify biological processes associated with the transcriptomic divergence between species. Pathways were ranked by −log_10_(false discovery rate; FDR), with higher values indicating stronger enrichment. The most significantly enriched pathway was ‘biosynthesis of secondary metabolites’ (egr01110; −log_10_FDR = 7.24). Based on fold enrichment (FE), four of the nine KEGG pathways passing the >1.5-FE cut-off were associated with secondary metabolism, including ‘biosynthesis of various plant secondary metabolites’ (egr00999; FE = 4.51) and ‘flavonoid biosynthesis’ (egr00941, FE = 3.99; Figure 3b). Other enriched pathways, including ‘tryptophan metabolism’, were associated with primary metabolic processes.

Broad pathways such as ‘biosynthesis of secondary metabolites’ contained the largest number of differentially expressed genes but showed lower FE, whereas more specific pathways generally contained fewer genes and higher FE. This pattern is consistent with enrichment of more specific specialized-metabolism pathways within the differentially expressed gene set. Inspection of these enriched pathways identified 16 putative methyltransferase genes distributed across secondary-metabolism-associated pathways, including ‘biosynthesis of secondary metabolites’, ‘phenylpropanoid biosynthesis’, and ‘flavonoid biosynthesis’ (Table S2).

### 2.3. Identification of Candidate O-Methyltransferases

We next sought to identify candidate OMTs potentially involved in flavonoid methylation. From the 16 differentially expressed putative methyltransferases identified in the transcriptomic analysis, candidates were assessed based on the presence of conserved Class II OMT motifs [9,30], methyltransferase-related gene annotations, and transcript abundance in leaves. Expression of eight putative OMTs was detected only in *E. eugenioides*, whereas the remaining eight were expressed in both species but differed in transcript abundance (Table S2). To prioritise candidates for functional testing, putative OMTs were ranked by transcript abundance in *E. eugenioides* leaves.

**Figure 4:**
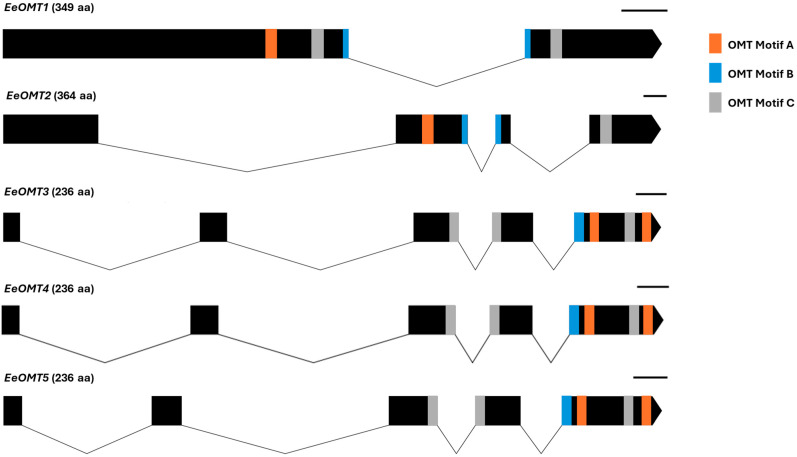
Gene structure of candidate *Eucalyptus* OMTs. Gene models were retrieved from the *E. grandis* reference genome (https://plants.ensembl.org/Eucalyptus_grandis/; accessed 4 March 2026). Protein lengths are shown in brackets. Black boxes represent exons, and coloured boxes indicate conserved Class II OMT motifs: Motif A, (V/I/L)(V/L)(D/K)(V/I)GGXX(G/A); Motif B, (V/I/F)(A/P/E)X(A/P/G)DAXXXK(W/Y/F); and Motif C, (A/P/G/S)(L/I/V)(A/P/G/S)XX(A/P/G/S)(K/R)(V/I)(E/I)(L/I/V); Motif assignments allowed up to two amino acid mismatches [9,30]. Scale bar = 100 bp.

Five candidate genes were prioritised for experimental studies (Table S3). *EeOMT3* and *EeOMT4* were selected for further characterisation because they were among the most highly expressed candidates (Table S2). *EeOMT5* was included because its translated protein sequence was highly similar to those of *EeOMT3* and *EeOMT4*, sharing 96% and 94% pairwise identity, respectively (Figure 4). *EeOMT1* (the closest homolog of *EnOMT1*) was also included. In parallel, the predicted structure of *En*OMT1, a previously characterised 7-OMT in *E. nitida* [25], was used to identify additional candidates with structural similarity to known eucalypt OMTs. AlphaFold-guided screening [31,32] identified 14 structurally similar proteins, with predicted local distance difference test (pLDDT) scores ranging from 79 to 94 (Table S4). One candidate, *EeOMT2*, showed a high pLDDT of 93 and its expression was detected in leaf RNA-seq data, so it was included for further characterisation.

### 2.4. Activity of Candidate Flavonoid O-Methyltransferase Activity of Candidate Enzymes

**Figure 5:**
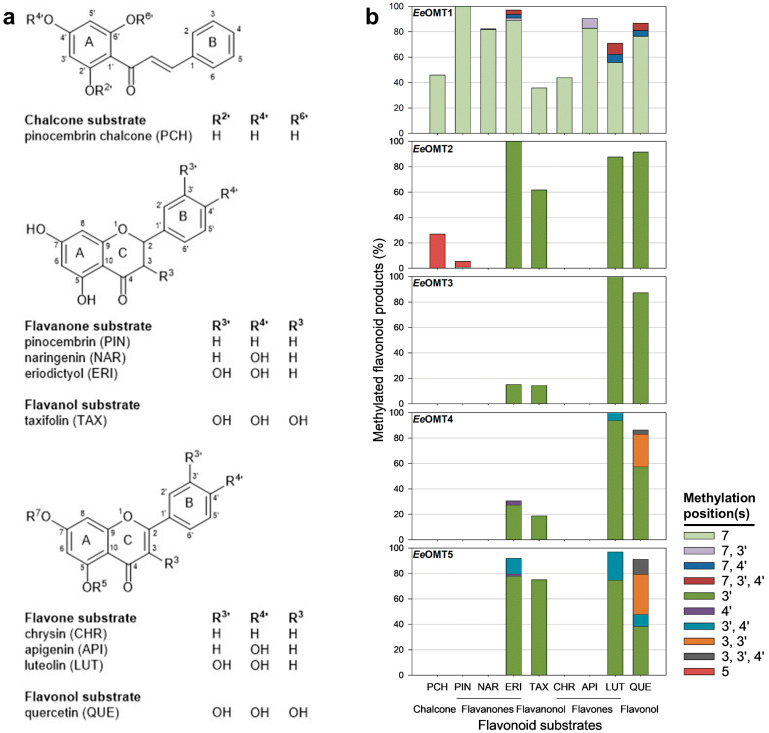
Flavonoid *O*-methyltransferase activity and substrate preferences of candidate OMTs. (**a**) Chemical structures of the flavonoids tested as substrates. (**b**) Relative methylation activity of recombinant *E. eugenioides* OMTs toward the flavonoid substrates. Activities are expressed as percentages and were normalised separately for each enzyme to the substrate showing the highest activity: *Ee*OMT1, pinocembrin; *Ee*OMT2, eriodictyol; *Ee*OMT3-5, luteolin. All substrates were assayed with a substrate concentration of 100 μM in the presence of excess SAM (500 μM). Descriptive statistics for the data are presented in Table S7. Abbreviations: PCH, pinocembrin chalcone; PIN, pinocembrin; NAR, naringenin; ERI, eriodictyol; TAX, taxifolin; CHR, chrysin; API, apigenin; LUT, luteolin; QUE, quercetin.

Coding sequences for the five selected OMT candidates were amplilfied from *E. eugenioides* leaf cDNA, cloned and heterologously expressed in *E. coli*. Partially purified enzyme preparations were assayed with SAM against a panel of flavonoid substrates with increasing degrees of hydroxylation: the flavanones pinocembrin, naringenin, eriodictyol; the flavanonol taxifolin; the flavones chrysin, apigenin and luteolin; and the flavonol quercetin (Figure 5a, Table S7).

The recombinant *E. eugenioides* OMTs exhibited distinct substrate preferences and methylation patterns. *Ee*OMT1 showed broad activity, catalysing *O*-methylation of all substrates tested (Figure 5b). Product profiles were dominated by 7-*O*-methylation, with minor amounts of di- and tri-methylated derivatives detected for eriodictyol, apigenin, luteolin, and quercetin. These di- and tri-methylated derivatives were all 7-*O*-methylated with additional methylation(s) at the 3′ or 4′ positions of the B-ring, or at both positions.

*Ee*OMT2 methylated the 5-hydroxyl of pinocembrin to produce alpinetin, although conversion was low under the assay conditions (4%). By contrast, *Ee*OMT2 methylated the equivalent 6′-hydroxyl of pinocembrin chalcone with higher conversion (27%). To assess whether *Ee*OMT2 could act on other A-ring-substituted flavanones, pinostrobin and cryptostrobin were tested as substrates. No methylation products corresponding to dimethylpinocembrin or 5-*O*-methylcryptostrobin were detected, suggesting that *Ee*OMT2 has narrow substrate specificity for A-ring methylation among the flavonoids tested. In addition to this A-ring/chalcone methylation activity, *Ee*OMT2 methylated the 3′-hydroxyl of the B-ring of flavanones and flavones in a strongly regioselective manner.

Liquid chromatography-mass spectrometry (LCMS) was used to characterise the product formed by *Ee*OMT2 from pinocembrin chalcone. The product showed an exact mass of *m*/*z* 271.0968 [M + H]^+^, consistent with the molecular formula C_16_H_14_O_4_ (calc. 271.0965). The 14 mass unit increase relative to pinocembrin chalcone (exact mass of *m*/*z* 257.0813 [M + H]^+^) is consistent with replacement of a hydroxyl proton with a methyl group. The product showed a broad UV absorption at 343 nm, characteristic of the B-ring cinnamoyl system of chalcones [33]. This distinguished it from the methylated flavanones alpinetin and pinostrobin, which showed narrower absorbances with λ_max_ of 285 and 289 nm, respectively. Methylation of pinocembrin chalcone by *Ee*OMT1 generated an isomeric product with the same exact mass but a later retention time and a slightly shifted UV λ_max_ at 339 nm. The *Ee*OMT2-derived methylated chalcone was chemically isomerised to alpinetin with acetic acid, and conversely, authentic alpinetin was subjected to the same acidic conditions and generated a compound matching the *Ee*OMT2 product’s retention time, UV spectrum and MS data. Together, the data support assignment of the *Ee*OMT2 product as alpinetin chalcone.

The other candidates, *Ee*OMT3–*Ee*OMT5, displayed narrower activity profiles. All three enzymes methylated substrates containing both 3′ and 4′-hydroxyl groups on the B-ring, but no detectable activity towards substrates containing only a 4′-hydroxyl group on the B-ring, such as naringenin and apigenin. *Ee*OMT3 exhibited highly specific activity towards the flavone luteolin and flavonol quercetin, with little detectable conversion of the flavanone eriodictyol or flavanonol taxifolin. It was the only enzyme in this study that methylated exclusively at one position, the 3′-hydroxyl of the B-ring. *Ee*OMT4 showed substrate preferences similar to *Ee*OMT3 but also formed di- and tri-methylated products, indicating capacity for sequential methylation at additional hydroxyl sites (Figure 5b). *Ee*OMT5 showed the broadest activity, converting B-ring hydroxylated substrates including eriodictyol, luteolin, taxifolin and quercetin, into mixtures of mono-, di- and tri-methylated products. Notably, the tri-methylated products generated by *Ee*OMT4 and *Ee*OMT5 included methylation at the 3-hydroxyl of the flavonol C-ring of quercetin, but not at the equivalent position in the flavanonol taxifolin.

### 2.5. EeOMT2 Enzyme Kinetics and Substrate Specificity

To examine the activity of *Ee*OMT2 towards pinocembrin and pinocembrin chalcone, enzyme kinetic assays were performed using affinity-purified protein, an excess of SAM (500 μM), and varying substrate concentrations under assay conditions optimized for each substrate. For pinocembrin chalcone, *Ee*OMT2 showed an apparent *K*_M_ value of 0.2 mM, an apparent *k*_cat_ of 0.049 min^−1^ and apparent *k*_cat_/*K*_M_ of 2.3 × 10^−4^ µM^−1^ min^−1^ (Table S8). By contrast, *Ee*OMT2 activity towards pinocembrin was barely detectable, and reliable kinetic parameters could not be measured. No activity was detected against pinostrobin chalcone or 2′,3′,4′-trihydroxychalcone, a pinocembrin chalcone isomer in which the 6′-hydroxyl is replaced by a 3′-hydroxyl.

### 2.6. EeOMT1/EeOMT2 Expression and O-Methylated Flavanone Accumulation

**Figure 6:**
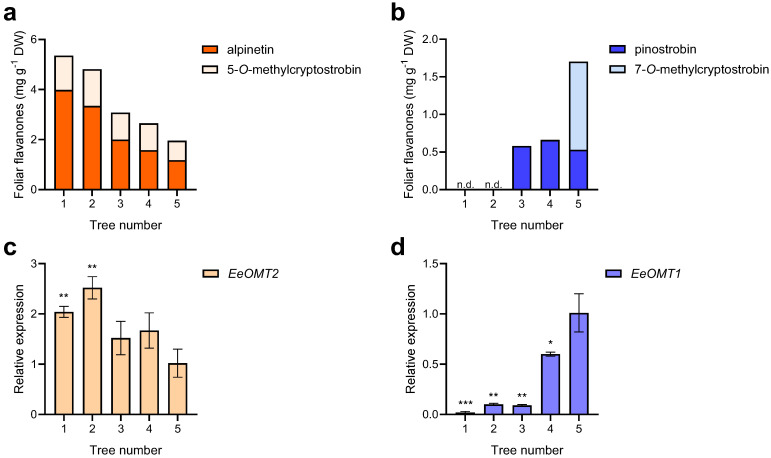
Leaf flavanone profiles and relative expression of *EeOMT1* and *EeOMT2* in five *E. eugenioides* trees. (**a**) Total abundance of 5-*O*-methylated flavanones, comprising alpinetin (ALP) and 5-*O*-methylcryptostrobin (5-*O*-CS), in leaves of five representative *E. eugenioides* trees. n.d., compound not detected. (**b**) Total abundance of 7-*O*-methylated flavanones, comprising pinostrobin and 7-*O*-methylcryptostrobin. (**c**,**d**) Relative transcript abundance of *EeOMT1* and *EeOMT2*, respectively, in leaves from the same trees. Metabolite abundances are expressed as mg g^−1^ of dry weight. Gene expression values are shown as mean ± SD from three independent replicates. Asterisks indicate statistically significant differences in gene expression, relative to tree 5, which was used as the calibrator: *p* < 0.05 (*), *p* < 0.01 (**), and *p* < 0.001 (***), using unpaired Student’s *t*-tests.

Variation in the accumulation of 5- and 7-*O*-methylated flavanones in leaves was observed across five *E. eugenioides* genotypes. We, therefore, examined whether transcript abundance of *EeOMT1* and *EeOMT2* was consistent with the accumulation of the corresponding *O*-methylated flavanone products (Figure 6). Relative expression of *EeOMT2* in leaves broadly corresponded with the abundance of 5-*O*-methylated flavanones, comprising alpinetin and 5-*O*-methylcryptostrobin. Trees 1 and 2 showed significantly higher *EeOMT2* transcript abundance and accumulated higher levels of 5-*O*-methylated products than tree 5, which showed the lowest abundance of these metabolites (Figure 6a,c). Similarly, increased *EeOMT1* expression was associated with the accumulation of 7-*O*-methylated flavanones. Tree 5, which accumulated the highest levels of 7-*O*-methylated compounds, showed the highest *EeOMT1* transcript abundance, whereas the remaining genotypes exhibited lower *EeOMT1* expression, and little to no accumulation of 7-*O*-methylated flavanones (Figure 6b,d). Together, these expression–metabolite patterns are consistent with proposed roles of *Ee*OMT2 and *Ee*OMT1 in the formation of 5-*O*- and 7-*O*-methylated flavanones, respectively, in *E. eugenioides*.

## 3. Discussion

This study identifies a set of *Eucalyptus O*-methyltransferases with distinct activities toward flavonoid substrates and provides evidence for a chalcone-stage methylation for the 5-*O*-methylated flavanone alpinetin. Comparative transcriptomics of two eucalypt species with contrasting flavanone profiles, combined with conserved OMT motif analysis, protein-structure-guided candidate selection and recombinant enzyme assays, identified five candidate OMTs that displayed divergent substrate preferences. Among these, *Ee*OMT2 was of particular interest because it methylated pinocembrin chalcone to form alpinetin chalcone, while showing relatively weak activity toward the corresponding flavanone, pinocembrin. These findings support a model in which at least some 5-*O*-methylated flavanones in eucalypts arise through methylation of chalcone precursors before flavanone ring closure by an as-yet-unknown chalcone isomerase.

The activity of *Ee*OMT2 provides a plausible biochemical solution to the problem of flavanone 5-*O*-methylation in eucalypts. This enzyme also catalysed direct methylation of pinocembrin to alpinetin but activity was barely detectable under the assay conditions used, consistent with previous reports of low-yield alpinetin formation by bacterial OMTs [27]. In flavanones, the 5-hydroxyl group forms a strong intramolecular hydrogen bond with the adjacent 4-carbonyl, which reduces its accessibility and alters its reactivity relative to other phenolic hydroxyl groups [34]. Methylation at the chalcone stage may avoid this constraint because the corresponding 6′-hydroxyl of pinocembrin chalcone (the equivalent position to the flavanone 5-hydroxyl) is more accessible for enzyme-catalysed deprotonation and methyl transfer. Consistent with this argument, *Ee*OMT2 methylated pinocembrin chalcone more efficiently than pinocembrin, and did not methylate 2′,3′,4′-trihydroxychalcone, which lacks the 6′-hydroxyl of pinocembrin chalcone.

**Figure 7:**
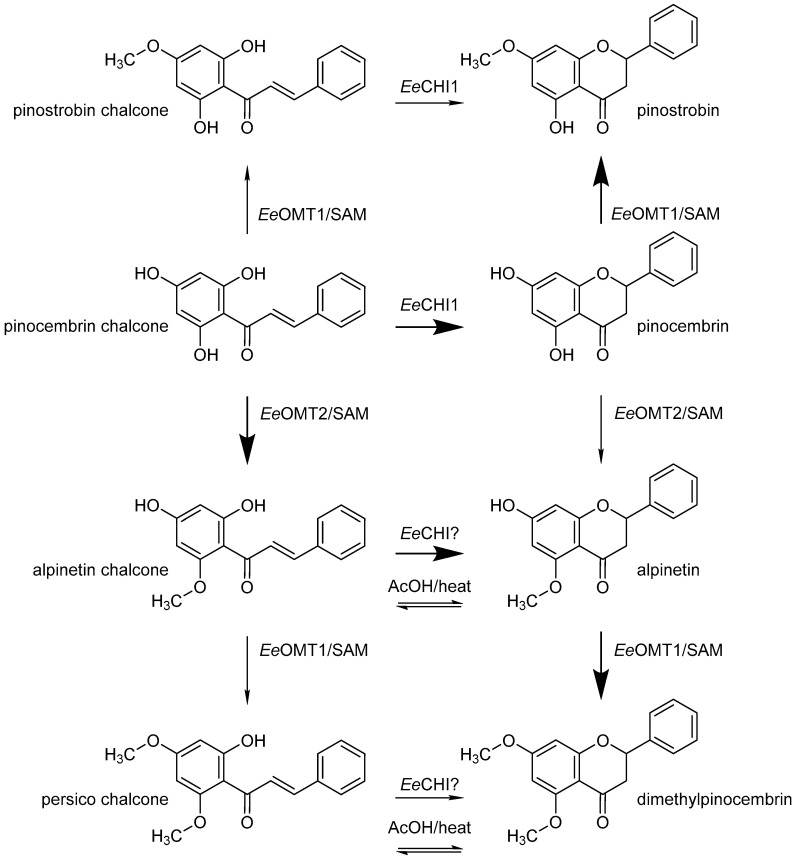
Proposed routes for *O*-methylated flavanone biosynthesis in eucalypts supported by this study. Larger arrows indicate proposed major routes. Chemical isomerisations of alpinetin chalcone and persico chalcone (flavokawain B) by AcOH/heat are shown; *in planta*, these conversions may require a distinct chalcone isomerase that has not yet been identified in eucalypts. Dimethylpinocembrin was produced by *Ee*OMT1-catalysed methylation of alpinetin, but not by *Ee*OMT2-catalysed methylation of pinostrobin. Similarly, *Ee*OMT1 converted alpinetin chalcone to persico chalcone, whereas *Ee*OMT2 did not methylate pinostrobin chalcone to this dimethylated product. These results suggest that dimethylpinocembrin biosynthesis may proceed via alpinetin or alpinetin chalcone intermediates in eucalypts, although the possible involvement of an independent dimethylating enzyme has not been excluded.

The low activity of *Ee*OMT2 on pinocembrin to form alpinetin directly is reminiscent of a previous study reporting that two Mg^2+^-dependent bacterial OMTs, StrAOMT and DesAOMT, produce alpinetin from pinocembrin with similar low efficiency [35]. Unlike *Ee*OMT2, however, these bacterial OMTs are Mg^2+^–dependent, and this metal cofactor can coordinate the carbonyl oxygen and the 5-hydroxyl oxygen of the substrate to stabilize the reaction intermediate. The predicted *Ee*OMT2 structure showed similarity to a plant flavanone CMT [28] and another eucalypt OMT, *En*OMT1 [25], which catalyses 7-*O*-methylation. Both *Ee*OMT1, the *E. eugenioides* homolog of *En*OMT1, and *Ee*OMT2 acted on pinocembrin chalcone, but they showed contrasting substrate preferences. *Ee*OMT1 was more active toward pinocembrin, consistent with 7-*O*-methylation of flavanones, whereas *Ee*OMT2 showed greater activity toward pinocembrin chalcone. Together with its expression in leaves, this is consistent with a role for *Ee*OMT2 in alpinetin biosynthesis *in planta* [35]. Based on this catalytic activity, we propose an alternative biosynthetic pathway for alpinetin (Figure 7).

*Ee*OMT2 exhibited a moderate apparent *K*_M_ value and low turnover on pinocembrin chalcone at 15 °C indicating a relatively low affinity for pinocembrin chalcone. Plant OMTs with similar chalcone 2′/6′-*O*-methylation activity have been characterised from *Medicago sativa* [36] and *Humulus lupulus* [37]. Considerably lower *K*_M_ values were reported for the *M. sativa* OMT against the deoxy chalcone isoliquiritigenin (2 µM) and for the *H. lupulus* OMT against the prenylated chalcone desmethylxanthohumol (18 µM). The relatively low apparent *K*_M_ value of *Ee*OMT2 could be attributed to the low temperature used for the bioassays to minimise the spontaneous isomerization of pinocembrin chalcone. Moreover, the kinetic properties are consistent with the relatively modest amounts of alpinetin observed in eucalypt leaves here (23% and 16% of total flavanones in *E. eugenioides* and *E. stenostoma*, respectively) and in the genus more broadly [14,15,16]. However, *in planta* abundance will also depend on precursor availability, competing pathway fluxes, enzyme expression and enzyme lifetime. Because pinocembrin chalcone is a common precursor to downstream flavanones, *Ee*OMT2 may encounter sufficient substrate during active flavonoid biosynthesis to support gradual accumulation of alpinetin and other 5-*O*-methylated flavanones during leaf expansion and maturation.

The conversion of alpinetin chalcone to alpinetin remains unresolved. Both *At*CHI2 from *Arabidopsis thaliana* [38] and *Ee*CHI1 (Table S3) converted pinocembrin chalcone to pinocembrin and pinostrobin chalcone to pinostrobin, but neither enzyme converted alpinetin chalcone to alpinetin under the conditions tested. This suggests that efficient conversion of alpinetin chalcone in eucalypts may require a distinct chalcone isomerase, an accessory factor, or favourable cellular conditions.

*Ee*OMT1 showed broad 7-*O*-methyltransferase activity across nine substrates within the flavonoid panel (Figure 5), irrespective of other ring substitutions, consistent with the previously characterised *E. nitida* homolog *En*OMT1 [25]. *Ee*OMT1 also 7-*O*-methylated alpinetin, alpinetin chalcone, and the *C*-methylated flavanones purified from eucalypts. Dimethylpinocembrin may, therefore, be biosynthesised by *Ee*OMT1 through 7-*O*-methylation of alpinetin, a second methylation step with experimental precedent from the activity of *En*OMT1 on alpinetin [25]. By analogy, 5-*O*-methyl cryptostrobin could arise through 6′-*O*-methylation of cryptostrobin chalcone by *Ee*OMT2, followed by chalcone isomerisation, although this remains to be experimentally demonstrated. Together, the activities of *Ee*OMT1 and *Ee*OMT2 provide a framework for proposing alternative routes to 7-*O*- and 5-*O*-methylated flavanones in *E. eugenioides*. These activities are consistent with the observed expression–metabolite associations across *E. eugenioides* genotypes, in which *EeOMT2* transcript abundance corresponded broadly with 5-*O*-methylated flavanone accumulation, while *EeOMT1* expression was associated with 7-*O*-methylated products.

Moving beyond chalcone and A-ring methylation, *Ee*OMT2–*Ee*OMT5 also catalysed flavonoid B-ring methylation. These enzymes methylated substrates containing adjacent 3′- and 4′-hydroxyl groups, with a preference for 3′-*O*-methylation (Figure 5). *Ee*OMT2 was the most regioselective, producing only 3′-*O*-methylated products, whereas *Ee*OMT4 and *Ee*OMT5 produced additional di- and tri-methylated products, indicating capacity for sequential methylation while favoring initial 3′-*O*-methylation. This preference is consistent with other plant flavonoid OMTs, including enzymes from *Glycine max* [39] and *Rhododendron delavayi* [28]. Unlike *Rd*OMT3, however, *Ee*OMT3 and *Ee*OMT5 retained activity toward substrates bearing a 4′-methoxy group, indicating differences in active-site tolerance among related flavonoid OMTs. The preference for 3′- over 4′-*O*-methylation was supported by the low activity towards the 3′-*O*-methylated flavonoids homoeriodictyol (3′-*O*-methyleriodictyol) and chrysoeriol (3′-*O*-methylluteolin), and by the ability of *Ee*OMT3 and *Ee*OMT5 to methylate the 4′-*O*-methylated flavonoids hesperetin (4′-*O*-methyleriodictyol) and diosmetin (4′-*O*-methylluteolin). Consistent with this regioselectivity, all three enzymes were unable to methylate flavonoids possessing only a 4′-hydroxyl group on the B-ring, such as the flavanone naringenin and flavone apigenin. These enzymes can therefore be considered primarily 3′-*O*-methylating OMTs.

The bicyclic core of flavones is planar due to the C-ring double bond, whereas flavanones are non-planar and chiral at C2 [40]. *Ee*OMT3 and *Ee*OMT4 preferentially methylated the flavone luteolin over the identically substituted flavanone eriodictyol (Figure 5). This preference for flavones is consistent with the flavone/flavanone substrate preference of four 3′-OMTs (*Gm*OMTs) identified from *G. max* [39]. *Ee*OMT5 showed weaker discrimination between these substrate classes.

*Ee*OMT4 and *Ee*OMT5 also catalysed limited methylation at the 3-hydroxyl of quercetin, but not at the equivalent position in the flavanonol taxifolin. Flavonol 3-*O*-methylation is noteworthy because methylation at this site is relatively uncommon in plants [41]. Most characterized flavonoid 3-OMTs specifically methylate the 3-hydroxyl and do not methylate hydroxyl groups on other rings. For example, *Rd*OMT10 from *R. delavayi* methylates the flavonols, kaempferol, quercetin, and isorhamnetin regiospecifically to their 3-*O*-methylated products [28]. By contrast, *Ee*OMT4 and *Ee*OMT5 showed sequential 3′/3-di-*O*-methylation activity on quercetin, suggesting broader substrate site tolerance.

*Ee*OMT3, *Ee*OMT4, and *Ee*OMT5 share 94–96% pairwise sequence identity, suggesting that their functional differences may arise from relatively small sequence variations. Their high sequence similarity is consistent with recent paralogy and functional divergence after gene duplication. Identifying the amino acid residues responsible for these differences may provide useful targets for engineering OMTs with tailored flavonoid methylation activities.

## 4. Materials and Methods

### 4.1. Plant Material

Newly developed, unexpanded leaves, approximately 2–3 weeks old were collected from selected *E. eugenioides* and *E. stenostoma* trees growing in a plantation in central Victoria, Australia (36.4641° S, 146.0213° E). For molecular analysis, up to 10 young leaves were immediately snap-frozen in liquid nitrogen and transported to the laboratory. For flavanone quantification and structural elucidation, bulk samples of fully expanded leaves, up to 100 leaves per tree, were collected from the same trees. Leaves were oven-dried at 50 °C for 72 h and ground using a mill (MF10 microfine grinder drive; IKA-Werke, Breisgau, Germany) fitted with a cutting/grinding head and a 1 mm sieve.

### 4.2. RNA Isolation and Expression Analysis (qRT-PCR)

*RNA* was isolated from leaf tissue (300–400 mg) using a CTAB-based method [42], with the following modifications. After chloroform extraction, an equal volume of isopropanol was added to the aqueous phase and the mixture was incubated at room temperature for 10 min, followed by centrifugation at 15,000× *g* for 20 min at 4 °C. The resulting pellet was washed with 600 µL of 70% (*v*/*v*) ethanol and resuspended in RNase-free water. RNA samples were further purified using the Spectrum Plant Total RNA Kit (Merck, Darmstadt, Germany) and treated with on-column DNase I, according to the manufacturer’s instructions. Approximately 1.5 µg of total RNA was used for first-strand cDNA synthesis with the Tetro cDNA Synthesis Kit (Bioline, London, UK), in a total reaction volume of 20 µL. Reactions were incubated at 45 °C for 30 min and were terminated by heating at 85 °C for 5 min. The resulting cDNA was diluted 1:16 for subsequent analysis. qRT-PCR analysis was performed using SYBR Green (Thermo Fisher Scientific, Waltham, MA, USA) according to the manufacturer’s instructions on a CFX384 Touch Real-Time PCR detection system (Bio-Rad Laboratories, Hercules, CA, USA). Oligonucleotide sequences are listed in Table S6. Cycling conditions were 95 °C for 2 min, followed by 30 cycles of 95 °C for 5 s, and 60 °C for 10 s. Melt curve analysis was performed using the real-time cycler’s built-in program. Relative expression levels were calculated using the 2^−ΔΔCT^ method [43], with *α-tubulin* as the housekeeping gene and *E. eugenioides* tree #5 as calibrator, set to a relative expression value of 1.

### 4.3. Transcriptome Sequencing

Total RNA samples from were obtained from pooled unexpanded leaves (approximately 10–15 leaves per genotype). For *E. eugenioides*, three independent biological replicates were analysed from which three independent library preparations and sequencing runs were generated. In the case of *E. stenostoma,* high quality RNA was obtained from only a single genotype, therefore, three independent libraries were prepared from the same pooled RNA sample prior to sequencing. Libraries were prepared using the Illumina’s TruSeq Stranded RNA library preparation kit and sequenced at the Australian Genome Research Facility (AGRF; Melbourne, Australia) on an Illumina NovaSeq X Plus RNA-seq platform, generating 150 bp paired-end reads. Raw reads were quality-checked and filtered to remove Illumina adapter sequences, overrepresented sequences, and potential cross-species contamination. Across all samples, >91% of bases had a Phred quality score ≥ Q30. Filtered reads were aligned to the *E. grandis* reference genome (GCA_000612305.1) using STAR (v2.3.5a). Read mapping rates and distribution across genomic features were summarized (Table S5). Transcript assembly was performed using StringTie version 2.1.4 [44] with the reference annotation-based transcript (RABT) option and the RefSeq genome annotation as a guide. Comparative transcriptomic analyses were conducted using edgeR version 4.0.9 in R [45]. Counts were normalized using the trimmed mean of M-values (TMM) method.

### 4.4. Cloning of Candidate OMTs

Full-length coding sequences of candidate OMTs (GenBank accession numbers: *Ee*OMT1_CDS PZ332309; *Ee*OMT2_CDS PZ332310; *Ee*OMT3_CDS PZ332311; *Ee*OMT4_CDS PZ332312; *Ee*OMT5_CDS PZ332313; *Ee*CHI1_CDS PZ332314) were amplified from *E. eugenioides* cDNA using 1.25 µL each of forward and reverse primers (10 mM; Table S6), 5 μL of 5× Phusion HF Buffer, 0.2 μL of Phusion HF polymerase (Thermo Fisher Scientific), 0.5 µL of 10 mM dNTPs, and 16.3 μL of nuclease-free water in a total reaction volume of 25 μL. PCR was performed under the following reaction conditions: 98 °C for 30 s, 35 cycles of 98 °C for 10 s, 55 °C for 30 s, and 72 °C for 30 s kb^−1^, followed by a final extension 72 °C for 5 min and 4 °C hold. Purified PCR products were digested with the appropriate restriction enzymes (*BamHI*, *EcoRI*, or *NotI*) and the pHUE vector, which contains an N-terminal 6 × His tag and a thrombin cleavage site, was linearised using the same enzymes with the addition of 1 μL TSAP (Promega, Madison, WI, USA). Following gel purification, T4 DNA ligations were set up for each candidate OMT according to the manufacturer’s instructions (Thermo Fisher Scientific), and 5 μL of each ligation reaction was transformed into *E. coli* DH5α cells. Plasmids were purified using the ZR Plasmid Miniprep Classic kit (Zymo Research, Irvine, CA, USA) and verified by Sanger sequencing at AGRF. Sequence-verified plasmids were subsequently transformed into *E. coli* BL21 (DE3) cells for enzyme assays.

### 4.5. Recombinant Protein Expression

Cells of *E. coli* BL21 (DE3) harbouring the expression vectors were grown in 100 mL LB medium supplemented with carbenicillin (100 μg mL^−1^) at 37 °C for approximately 5 h, until the culture reached an OD 600 of 0.6. Protein production was induced by the addition of 0.1 mM isopropyl β-D-1-thiogalactopyranoside and cultures were incubated at 16 °C for 16 h [25]. Cells were harvested by centrifugation (3000× *g* for 5 min) and the pellet was resuspended in lysis buffer (50 mM NaH_2_PO_4_, 300 mM NaCl, 10 mM imidazole), supplemented with protease inhibitor cocktail (Roche, Basel, Switzerland) and 1 mg mL^−1^ Lysozyme (Thermo Fisher Scientific). The suspension was gently mixed at room temperature for 30 min and sonicated to release intracellular content. The lysate was centrifuged twice at 10,000× *g* for 5 min, and the clarified supernatant was used as crude enzyme extract for the enzyme assays.

### 4.6. Enzyme Assays and Kinetics

Enzyme activity of crude extracts was assayed as described [25], with the following modifications: reaction mixtures contained 50 mM sodium phosphate buffer (pH 7), 200 μL of crude OMT enzyme (approx. 0.6 mg of total protein), 500 μM *S*-adenosyl-L-methionine (SAM), and 100 μM substrate dissolved in DMSO, in a total reaction volume of 500 µL. Reactions were incubated at 30 °C with gentle shaking for 2 h and terminated by the addition of an equal volume of acetonitrile. The solvent fraction of the assay mixture containing methylated products was separated overnight at −20 °C and subsequently analysed by HPLC. Negative controls (reactions lacking enzyme, substrate or SAM) were included alongside experimental samples to ensure analytical accuracy. All authentic flavonoid standards used as substrates and to identify methylated products were purchased from Extrasynthese (Genay, France).

*Ee*OMT2 was purified with immobilized metal affinity chromatography and quantified with bicinchoninic acid (BCA) assay for enzyme kinetic assays. Enzyme kinetic parameters of *Ee*OMT2 were calculated using pinocembrin chalcone and pinocembrin as substrates. The enzyme kinetics assays were performed with the same procedure mentioned above with the following modifications: reaction mixtures contained 50 mM sodium phosphate buffer (pH 5.5), 300 μL of crude *Ee*OMT2 enzyme (approx. 0.9 mg of total protein), and 37.5 μL of SAM (10 mM) in a total reaction volume of 750 μL at 15 °C. Duplicate assays and varying substrate concentrations (9.8–588 µM) at a saturating concentration of SAM were carried out for time intervals ranging from 15 to 40 min, where the reaction velocity is in the linear range with respect to time for *Ee*OMT2 with pinocembrin chalcone and pinocembrin as substrates. The catalytic rate constant (*k*_cat_) was determined by performing a short assay with a substrate concentration at *K*_M_ using an enzyme purified through His-tag affinity chromatography. The kinetic constants (*V*_max_ and *K*_M_) were then calculated using the Enzyme Kinetics add-on in SigmaPlot version 16 (Grafiti LLC, Palo Alto, CA, USA) by fitting data to the single substrate, Michaelis–Menten equation. K_M_ was averaged between duplicate experiments (Table S8).

### 4.7. Chemical and Enzymic Rearrangement of Chalcones to Flavanones

Alpinetin chalcone, the methylated product produced by *Ee*OMT2 on pinocembrin chalcone, was subjected to chemical isomerisation to alpinetin. The product mixture was dried and reconstituted in glacial acetic acid (300 μL). The solution was then heated at 70 °C for 16 h. Following incubation, the mixture was dried and redissolved in acetonitrile (300 μL) prior to HPLC analysis. The same protocol was also applied to rearrange an authentic standard of alpinetin into alpinetin chalcone.

The coding sequence of a chalcone isomerase (chalcone-flavanone isomerase; *AtCHI2*; UniProt: Q9FKW3) from *Arabidopsis thaliana* was *de novo* synthesised as a gene block (Twist Bioscience, San Francisco, CA, USA) to assess its activity toward the flavanone chalcone substrates pinocembrin chalcone, pinostrobin chalcone and alpinetin chalcone. The *AtCHI2* CDS sequence included restriction site overhangs (*BamHI* and *NotI*) enabling cloning into the pHUE expression vector. The candidate *EeCHI1*, identified through AlphaFold-guided homology searches using the *At*CHI2 protein sequence as a query, was selected based on its expression in leaves and subsequently isolated and cloned from *E. eugenioides* cDNA using the primers described in Table S6. Recombinant protein expression was carried out as described in Section 4.5, with the modification of reducing temperature to 15 °C to minimise possible spontaneous rearrangement of chalcones to their respective flavanones [46].

### 4.8. HPLC and LCMS

HPLC was performed using a Prominence system (Shimadzu Corporation, Kyoto, Japan) fitted with a Phenomenex Gemini C18 column (Danaher, Washington, DC, USA) eluted at a flow rate of 1.0 mL min^−1^ [19]. LCMS analysis was performed using an Agilent Revident 6520 accurate-mass QTOF mass spectrometry system (Agilent Technologies, Santa Clara, CA, USA), with a dual electro-spray ionisation source attached to an Agilent 1200 Infinity HPLC with a diode array detector using the same column and eluent system as the HPLC, but with a flow rate of 0.8 mL min^−1^. The LCMS was run in both positive and negative modes with an ion-source voltage of 150 V (CID of 10).

Flavonoids in leaf extracts and bioassays were identified and quantified based on comparison to authentic standards (Extrasynthese) or compounds purified in-house and using the characteristic LCMS fragmentation patterns of flavonoids [47,48]. In addition, the identities of 7-*O*-methylcryptostrobin, 7-*O*-methyldemethoxymatteucinol, and 5,7-di-*O*-methylcryptostrobin were confirmed by the action of the recombinant protein of *En*OMT1 on cryptostrobin, demethoxymatteucinol and 5-*O*-methylcryptostrobin substrates, respectively.

### 4.9. Nuclear Magnetic Resonance (NMR) Spectroscopy

NMR spectra were collected on a Bruker Avance spectrometer (Bruker Corporation, Billerica, MA, USA), ^1^H NMR 500 MHz and ^13^C NMR 125 MHz. Compound 1 was dissolved in methanol-d_4_ (MeOD), and compound 2 was dissolved in chloroform-d_1_ (CDCl_3_). Spectra were referenced to the residual solvent peaks (MeOD: δ 3.31 ppm and δ 49.00 ppm; CDCl_3_: δ 7.26 ppm and δ 77.16 ppm). Chemical shifts (δ) are reported in parts per million (ppm) and coupling constants (J) reported in Hz. Multiplicities are reported as follows: s, singlet; d, doublet; dd, doublet of doublets; t, triplet; sept, septet; m, multiplet (Appendix A).

### 4.10. X-Ray Diffraction

X-ray diffraction data (Appendix A) were collected on a Rigaku Synergy S X-ray diffractometer (Rigaku Corporation, Tokyo, Japan) equipped with a Cu Kα sealed X-ray tube. Cell refinement and data reduction were performed in CrysAlisPro [49]. Structures were refined in WinGX [50] using the SHELX suite [51].

## 5. Conclusions

In support of our hypothesis, functional characterisation of eucalypt OMTs revealed a chalcone OMT, *Ee*OMT2, that produces alpinetin chalcone, a plausible direct biosynthetic precursor of alpinetin. These findings support an alternative pathway in which 5-*O*-methylated flavanones are formed through methylation of chalcone precursors before flavanone formation, rather than solely through direct methylation of flavanones. Understanding the biosynthesis of alpinetin and related compounds may guide efforts to enhance production of methylated flavanones in plant-based products or produce high-value metabolites via synthetic biology. Other candidate OMTs also exhibited distinct flavonoid methylation activities. Broad-spectrum enzymes such as *Ee*OMT1 may enable the production of diverse methylated flavonoids in heterologous systems, while highly selective enzymes such as *Ee*OMT2–*Ee*OMT5 provide tools for controlled flavonoid scaffold modification, particularly with respect to A-, B-, and C-ring methylation and substrate preferences for flavanones, flavanonols, flavones, or flavonols. Complementary downstream assays are key to determining how these methylation patterns influence pharmacological and therapeutic properties. Future work should focus on heterologous pathway reconstruction, structural validation of OMT/substrate interactions, and *in planta* functional analysis through transient expression or knockouts approaches.

## Data Availability

Sequence data have been submitted to GenBank and are available from the corresponding author upon reasonable request pending public release. CCDC 2453271 contains the X-ray diffraction crystallographic data for this paper. These data can be obtained free of charge via www.ccdc.cam.ac.uk/data_request/cif, (accessed 27 March 2026) or by emailing data_request@ccdc.cam.ac.uk, or by contacting the Cambridge Crystallographic Data Centre, 12 Union Road, Cambridge CB2 1EZ, UK; fax: +44-1223-336033.

## Supplementary Materials

The following supporting information can be downloaded at: https://www.mdpi.com/article/10.3390/ijms27115078/s1.

## Abbreviations

The following abbreviations are used in this manuscript:

CMT	*C*-methyltransferase
DEGs	Differentially expressed genes
FDR	False discovery rate
FE	Fold enrichment
HPLC	High performance liquid chromatography
KEGG	Kyoto encyclopedia of genes and genomes
LCMS	Liquid chromatography-mass spectrometry
NMR	Nuclear magnetic resonance spectroscopy
OMT	*O*-methyltransferase
PCR	Polymerase chain reaction
pLDDT	Predicted local distance difference test
qRT-PCR	Quantitative reverse transcription PCR
SAM	*S*-adenosyl-L-methionine

## Appendix A. Structural Elucidation of C-Methylated Flavanones

Two putative flavanones in the eucalypt leaf extracts could not be definitively identified by comparison to available commercial or in-house flavanone standards. Their MS data were identical, with an exact mass of *m*/*z* 285.1103 [M + H]^+^, corresponding to the molecular formula C_17_H_16_O_4_ (calcd for [M + H]^+^, 285.1127), consistent with dimethylated flavanones. To identify the compounds, bulk acetonitrile extraction of *E. eugenioides* leaves (100 g) was performed, and the extract was fractionated using a Reveleris X2 medium-pressure liquid chromatography system fitted with a Büchi FlashPure Select C-18 cartridge (4 g; Büchi Labortechnik, Flawil, Switzerland), using the same mobile phases as for HPLC. Both compounds were obtained as white crystals.

The ^1^H NMR spectrum of compound 1 showed two methyl singlets at δ 2.01 ppm (3H, s, *C*-Me) and 3.80 ppm (3H, s, *O*-Me), indicating the presence of *C*-methyl and *O*-methyl groups (Table S9). Four possible isomers are consistent with this methylation pattern, and the NMR spectra alone did not allow unambiguous assignment. Compound 1 was therefore crystallized, and its structure was determined by single-crystal X-ray diffraction. Crystals were grown by slow diffusion of n-hexane into ethyl acetate, and intensity data were collected on a Rigaku Synergy S diffractometer (Rigaku Corporation, Tokyo, Japan) using Cu Kα radiation. Crystal data: C_17_H_16_O_4_, Mr = 284.30, *T* = 100 K, λ = 1.54184 Å, monoclinic, space group C2, *a* = 23.0977(5) Å, *b* = 5.12320(10) Å, *c* = 24.3060(5) Å, α = 90°, β = 99.940(2)°, γ = 90°, *V* = 2833.05(10) Å^3^, *Z* = 8, Dc = 1.333 g cm^−3^, µ = 0.778 mm^−1^ (Cu), F(000) = 1200, crystal size = 0.101 × 0.050 × 0.034 mm^3^. A total of 16,601 reflections were measured to θmax = 79.134°, yielding 5920 independent reflections [R(int) = 0.0764]. Final refinement gave R = 0.0502 and wR(F2) = 0.1207 [I > 2σ(I)], with goodness of fit = 1.071. The absolute structure parameter was 0.06(17). CCDC: 2453271. X-ray diffraction established the structure of compound 1 as 5-*O*-methyl cryptostrobin with an (*S*) configuration (Figure S1). The NMR data for compound 1 were in good agreement with a previous report of this compound [52].

The ^1^H NMR spectrum of compound 2 showed two methyl singlet signals at δ 2.01 ppm (3H, s, H-11) and 3.84 ppm (3H, s, H-12), consistent with a *C*-methyl group and an *O*-methyl group (Table S9). The HMBC spectrum showed correlations from both methyl signals to the same carbon signal at δ_C_ 165.77 ppm (C-7), indicating that the methyl and methoxy groups are ortho to each other. In addition, the *C*-methyl singlet signal at δ_H_ 2.01 ppm and the H-2 signal at 5.42 ppm showed HMBC correlations to the same carbon signal at δ_C_ 161.16 ppm (C-9), consistent with the attachment of the *C*-methyl group to C-8. This assignment places the methoxy group at C-7. Compound 2 was, therefore, assigned as 7-*O*-methyl cryptostrobin, and its NMR data were in good agreement with literature [53].
