# Supplementary material for: Flavonoid O-Methyltransferases in Eucalyptus—Biosynthesis of Alpinetin via a Methylated Chalcone Precursor
Source: Int J Mol Sci. 2026 Jun 4;27(11):5078. doi: 10.3390/ijms27115078 (PMC13256973; doi:10.3390/ijms27115078)
Supplement: Supplementary file 1 [file ijms-27-05078-s001.zip › ijms-4318774-supplementary.pdf]

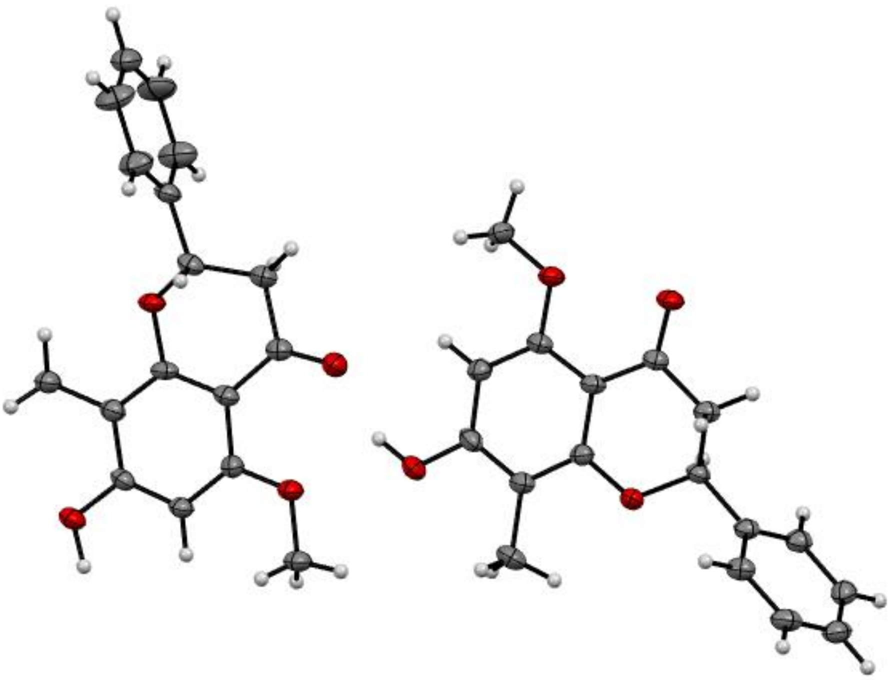

Figure S1. ORTEP representation of the asymmetric unit of compound 1 (5-*O*-methylcryptostrobin), as determined by X-ray diffraction. Thermal ellipsoids are shown at the 50% probability level.

Table S1. Foliar flavanone profile data for *E. eugenoides* and *E. stenostoma*. Abbreviations: DW, dry weight; SE, 1 standard error of the mean; nd, not detected; PIN, pinocembrin; ALP, alpinetin; PIS, pinostrobin; DMP, dimethylpinocembrin; 5CS, 5-*O*-methylcryptostrobin; 7CS, 7-*O*-methylcryptostrobin; 7DM, 7-*O*-demethoxymatteucinol; CSB, cryptostrobin; DMM, demethoxymatteucinol.

**Species**		**Flavanones**								
		PIN	ALP	PIS	DMP	5CS	7CS	7DM	CSB	DMM
E. eugenioides $n = 5$	Mean (mg g^{-1} DW)	2.75	6.67	0.32	5.52	2.42	0.63	0.26	9.06	0.68
	SE	0.55	1.93	0.07	1.90	0.55	0.28	0.03	0.88	0.05
	Minimum	0.85	3.04	0.20	0.56	0.70	0.26	0.16	6.87	0.53
	Maximum	3.96	13.38	0.58	12.06	4.19	1.72	0.31	11.87	0.77
E. stenostoma $n = 2$	Mean (mg g^{-1} DW)	12.56	3.12	0.34	2.69	nd	nd	nd	nd	nd
	SE	2.39	1.15	0.03	0.26	nd	nd	nd	nd	nd
	Minimum	10.17	1.97	0.31	2.43	nd	nd	nd	nd	nd
	Maximum	14.95	4.27	0.37	2.95	nd	nd	nd	nd	nd

Table S2. Putative methyltransferases (n = 16) identified through differential gene expression analyses comparing *E. eugenioides* and *E. stenostoma*. Candidate genes were ranked according to the likelihood ratio (LR) from the edgeR generalized linear model likelihood ratio test (LRT), which indicates the strength of evidence for differential expression. Putative methyltransferases with highest expression in *E. eugenioides* are highlighted in grey. The selected candidate OMTs EUGRSUZ_G01610, EUGRSUZ_H00347 and EUGRSUZ_A01392 were cloned and heterologously expressed, but did not exhibit methylation activity against the panel of flavonoid substrates tested.

Gene (*E.grandis*)	Annotation (*E.grandis*)	log_2FC	log_2CPM	LR	p-value	FDR	*E. eugenioides* raw read counts	*E. stenostoma* raw read counts
EUGRSUZ_G01610	S-adenosyl-L-methionine-dependent methyltransferases superfamily protein	16.23	8.04	144.55	2.69E-33	1.78E-29	14,333	0
EUGRSUZ_A01392	O-methyltransferase 1	13.22	6.27	67.09	2.59E-16	1.98E-13	1,501	0
EUGRSUZ_E03339	O-methyltransferase 1	10.95	2.50	37.58	8.79E-10	1.70E-07	349	0
EUGRSUZ_A01389	O-methyltransferase 1	11.95	5.13	36.99	1.19E-09	2.17E-07	462	0
EUGRSUZ_G00017	O-methyltransferase 1	10.61	4.38	28.65	8.68E-08	8.54E-06	235	0
EUGRSUZ_H04650*	caffeoyl-CoA 3-O-methyltransferase	3.77	5.60	28.25	1.07E-07	1.01E-05	3,503	89
EUGRSUZ_H04646*	caffeoyl-CoA 3-O-methyltransferase	5.09	3.96	24.59	7.10E-07	4.80E-05	1,109	11
EUGRSUZ_E03148	O-methyltransferase 1	9.31	0.93	23.85	1.04E-06	6.47E-05	110	0
EUGRSUZ_A01395	O-methyltransferase 1	4.62	6.14	21.78	3.05E-06	1.60E-04	968	30
EUGRSUZ_I02810	O-methyltransferase 1	9.53	3.31	19.82	8.51E-06	3.56E-04	120	0
EUGRSUZ_E03146	O-methyltransferase 1	8.10	-0.20	19.59	9.61E-06	3.94E-04	61	0
EUGRSUZ_G00016	S-adenosyl-L-methionine-dependent methyltransferases superfamily protein	-2.92	0.91	16.58	4.66E-05	1.37E-03	16	88
EUGRSUZ_C00903	S-adenosyl-L-methionine-dependent methyltransferases superfamily protein	-1.73	4.75	15.02	1.07E-04	2.60E-03	410	710
EUGRSUZ_H00347	O-methyltransferase 1	3.21	6.67	14.94	1.11E-04	2.68E-03	6,481	224
EUGRSUZ_K02257	S-adenosyl-L-methionine-dependent methyltransferases superfamily protein	3.28	1.32	13.82	2.01E-04	4.29E-03	128	7
EUGRSUZ_C00925	S-adenosyl-L-methionine-dependent methyltransferases superfamily protein	3.75	3.99	13.82	2.02E-04	4.29E-03	415	8

*Putative homologs of EeOMT3 (EUGRSUZ_H04650) and EeOMT4 (EUGRSUZ_H04646) in *E. grandis*.

Table S3. Nucleotide coding sequences and deduced amino acid sequences of candidate *O*-methyltransferases (OMTs) and an isomerase identified and cloned from *E. eugenioides* leaves.

Gene name	Nucleotide coding sequence	Amino acid sequence
EeOMT1	ATGGCACCGCTCGATGAAACGTTAAGTGGCCAAG<br>TCCAAGTATGGAAGCTCATGTATGCGTACGTGGAC<br>TCAATGGCGCTGAACTGCGCGGTGGAGCTCCGGA<br>TACCTGACATCATCCACTCGCTGGGCGGGGGGCC<br>TGTCACCTTGGCTCAAATAGCCTCCCACATCCCCT<br>CCCCATCTGTCAAAACCTGCGTCCTCGGCCGCATC<br>ATGACGCCCCTGGTCCGCAAGAACATCTTCTCCG<br>CGCACTACGACGGCAGAGAGACGCTCTACGGCCT<br>CACCCCGTCGTCGAGATGGCTCTTACAAGGGGCT<br>GGGCACCTCAACCTCGCGCCACTGGTACTGCTGG<br>CGGGCCATCAGTATATGGTGTCCCCGTGGCACAG<br>CCTTAGTGACTACATCAAGGACGGGGGATCATTC<br>CCGTTAAAGAGGGCTTATGGCTGCGAAATGTGGG<br>ACTTGGAGTCCCAGAACCCTGAATTCAGCCGCAC<br>GTTCCGTGATGCAATGGCGTGCTCGAACAAGCTC<br>ATGATGAAAGCTATCGTGGATGCGTACAAGGATG<br>GGTTCGAGCGCGTGGGATCTCTGGTGGACGTTGG<br>AGGCGGGACGGGCAGTGCCGTAACCGAGATAGT<br>GAGGGCATACCCGCACATCAAGGGGATTAACTTC<br>GACCAGCCTCACATTGTTGCTGCCGCGCCAGCGC<br>ACGGCAGGGTCAGTCATGTCGGGGGAGACATGTT<br>TGAGGCCATTCCAAGTGCTGATGCTGTTTTCATGA<br>AGTGGATCCTGCATGATTGGAATGATGAAGATTCT<br>GTAAGGATTTTGAAGAACTGCAGGAGAGCTGTAG<br>CGGAAAAGAATGGCAAGGTGATCCTGGCGGAAG<br>TTGTGTTGCGACCAGAGGGCGATGGCCTCTTCGAT<br>GACACGGGGATAGCTTCCGATCTGACCATGATCA<br>CTCAAACCGGAGGTAAAGAGAGGACCGAACCAG<br>AGTGGAAGAAGCTTTTGGAGGAAGGAGGCTTCCC<br>CCGCTGCAATATCTTCCAAACCCCTTCCTTGTTGTC<br>CATCATTGAAGCCTTCCCAGCTTGA	MAPLDEMLSGQVQVWKLMYAYV<br>DSMALNCAVELRIPDIIHSLGGGPV<br>TLAQIASHIPSPAIKTCVLGRIMTPL<br>VRKNIFSAHYDGRETLYGLTPSSRW<br>LLQGAGHLNLAPLVLLAGHQYMV<br>SPWHSLSDYIKDGGSFPLKRAYGCE<br>MWDLESQNPEFSRTFRDAMACSN<br>KLMMKAIVDAYKDGFEHVGSLVD<br>VGGGTGSAVAEIVRAYPHIKGINFD<br>QPHIVAAAPAHGGVSHVGGDMFE<br>AIPSADAIFMKWILHDWNDEDSVR<br>ILKNCRRAVAEKNGKVILAEVVLR<br>PEGDGLFDDTGIASDLTMITQTGG<br>KERTEPEWKKLLEEGGFPRCNIFQT<br>PSLLSIIEAFPA*

EeOMT2	ATGGCCGACAACCAAGAACGCGAAGGGCGCGAT<br>CAAGAAGAGGAAGTCGGGAAGCTGGCGGTCCGG<br>CTGGCCAGCTCGGTGGTGCTCCCGATGACCCTCA<br>AGTCGGCCCTCGAGCTCGGTATCATCGACGCCCTC<br>GTCTCCACCGGTGGGTTCCTCTCGGCTGCCGAGAT<br>AGCGAGCCGGGTCGGCGCCAAGAACCCGGGGGC<br>CCCGGTCCTGGTGGACCGGATGATGCGCCTCCTG<br>GCGAGCCACGGCGTGATCGAGTGGCGGTCGAGG<br>AGGGGCGACGGCGACGGAGATGGGGGGGAGAG<br>AGAGTACGGTCCGGGACCCATGTGCAGGTTCTTT<br>GCCAAGGACGAAGAAGGTGGAGCTGTTGGTCCTC<br>TGTTTCTGCTGCATCACGACAAGGTCTTCATGGAG<br>AGTTGGTACCACTTGAACGATGTCATCATGGAAG<br>GAGGGGTTCCGTTTGAGAGGGTATACGGGGTGAC<br>GGCATTCGAGTATCCCGCCATTGACGACAGGTTC<br>AATCAAGTTTTCAACCGAGCCATGGCGAGTCATA<br>CTTCCTTGGTCATGAAGAAAATACTCGATGTCTAC<br>AGAGGGTTCGAAGGCATCAAAGTGCTGGTCGATG<br>TGGGAGGCGGAGTCGGGGTCACTCTCAAGATGAT<br>CACCTCCAAGCATCCCCACATCAAGGGCATCAAC<br>TTCGACTTGCCTCACGTCTTGGCCGATGCTCCTTCT<br>TATCCAGGTGTCGAGCGTGTTGGTGGAGATATGTT<br>TGAGAGTGTTCCTACAGGAGATGCCATTTTCATGA<br>AGTGGATACTCCACGATTGGAGTGATGAGCATTG<br>CTCAAAACTTCTAAAGAACTGTTTTGAGGCTTTGC<br>CTGCCAATGGGAAGGTGATCATCGTCGAGGCGAT<br>TCTCCCCGTGGTTCCAGAGAGAGATGTCTCTTCAA<br>ACATTGTGTTCCAGCAAGACCTCTTCATGCTGGCT<br>CAAAATCCCGGCGGTAAAGAGAGGACGCAGAAG<br>GAGTATGAGGCCCTGGCAGTGCAGGCGGGATTCA<br>CCGGCTGTGAAGTCAAGTGCTGCGCTTACAACAG<br>CTGGGTAATGGAGTTCCCCAAAAAGGCAGGTCAT<br>TAA	MADNQEREGRDQEEEVGKLAVRL<br>ASSVVLPMTLKSALELGIIDALVST<br>GGFLSAAEIASRVGAKNPGAPVLV<br>DRMMRLLASHGVIEWRSRRGDGD<br>GDGGEREYGPGPMCRFFAKDEEG<br>GAVGPLFLLHHDKVFMESWYHLN<br>DVIMEGGVPFERVYGVTAFEYPAID<br>DRFNQVFNRAMASHTSLVMKKIL<br>DVYRGFEGIKVLVDVGGGVGVTLK<br>MITSKHPHIKGINFDLPHVLADAPS<br>YPGVERVGGDMFESVPTGDAIFMK<br>WILHDWSDEHCSKLLKNCFEALPA<br>NGKVIIVEAILPVVPERDVSSNIVFQ<br>QDLFMLAQNPGGKERTQKEYEAL<br>AVQAGFTGCEVKCCAYNSWVMEF<br>PKKAGH*
EeOMT3	ATGGAGCGAGGCTGGGACAAGGGCGAGATCCTG<br>GCAAGCAAAGCTCTCTCGAAGTACATATTGGAGA<br>CGAATGCATATCCAAGAGAGCACGAGCAGCTAAA<br>AGAACTCAGGGAGGCCACGGTCCAGAAGTACCA<br>AATCCGGAGTATAATGAACGTGCCGGCTGATGAG<br>GGGCAGCTAATCTCCATGATGTTGAAGCTCATGAA<br>TGCAAAGAAAACAATCGAGATCGGAGTCTTCACG<br>GGCTATTCTCTTCTCACCACCGCACTTGCACTTCC<br>GGCCGACGGCAAGATAATAGCGATAGACCCGGAT<br>AAGGAGGCCTATGAAATTGGCCTGCCATATATCCG<br>AAAAGCCGGAGTCGATCATAAGATCAACTTCATC<br>CAATCGGATGCTTTCTTGGTTCTGAACGACCTTAT<br>CGCGGATAGCCAAGAAGAGGGGACCTTTGATTTT<br>GCTTTCGTGGATGCCAAGAAGGACGACTACATGA<br>AATACCACGAGCTGGTGCTCAAGCTGGTGAAGGT<br>CGGAGGAGTGATCGGGTACGACGACACCCTGTGG<br>TTTGGTGCGGTCGCGCTCTCCGAAACCGACGAGA<br>TGGGCGACCACCACAAGCTCTGGAGAGACCAGC<br>TCAGGGAGTTCAACAGCTTCGTGGCGAAGGATCC	MERGWDKGEILASKALSKYILETN<br>AYPREHEQLKELREATVQKYQIRSI<br>MNVPADEGQLISMMLKLMNAKK<br>TIEIGVFTGYSLLTTALALPADGKII<br>AIDPDKEAYEIGLPYIRKAGVDHKI<br>NFIQSDAFLVLNDLIADSQEEGTFD<br>FAFVDAKKDDYMKYHELVLKLVK<br>VGGVIGYDDTLWFGAVALSETDEM<br>GDHHKLWRDQLREFNSFVAKDPR<br>VECCLLSVGGGLTLCRRLY*

	GCGTGTCGAGTGCTGTCTTCTTTCTGTCGGAGGTG<br>GCCTCACGCTTTGTAGGCGCCTCTATTAG	
EeOMT4	ATGGAGCAAGGCTGGGACAAGGGCGAGATCCTG<br>GCAAGCAAAGCTCTCTCGAAGTACATATTGGAGA<br>CGAATGCATATCCGAGAGAGCACGAGCAGCTGA<br>AAGAACTCAGGGAGGCCACAGTCCAGAAGTACC<br>AGATCCGGAGTATAATGAACGTGCCGGCTGATGA<br>GGGGCAGCTGATCTCCATGATGTTGAAGCTCATG<br>AATGCGAAGAAGACGATCGAGATCGGAGTCTTCA<br>CCGGCTACTCTCTTCTCACCACCGCACTTGCACTT<br>CCGGCCGACGGCAAGATAACAGCGATAGACCGG<br>GATAAGGAGGCCTATGAAATTGGCCTGCCATATAT<br>CCGAAAAGCCGGAGTTGATCATAAGATCAACTTC<br>ATCCAATCGGATGCTTTCTCGGTTCTGAATGACCT<br>CATCGCGGATAGCCAAGAAGGGGGGACCTTTGAC<br>TTTGCTTTCGTGGATGCCAAGAAGGACGACTTCAT<br>GAAGTACCACGAGCTGGTGCTCAAGCTGGTGAAG<br>GTCGGAGGTGTGATCGGGTACGACGACACCCTGT<br>GGTTTGGTGCGGTCGCACTCTCCGAAACCGATGA<br>CATGGGCGACCACCTGAAGCTCTGGAGAGACCA<br>ACTCAGGGAGTTCAACAGCTTCCTGGGGAAGGAT<br>CCACGTGTCGAGTGCTGTCTTCTTTCTGTCGGAGG<br>TGGCCTCACGCTTTGTAGGCGCCTCTATTAG	MEQGWDKGEILASKALSKYILETN<br>AYPREHEQLKELREATVQKYQIRSI<br>MNVPADEGQLISMMLKLMNAKK<br>TIEIGVFTGYSLLTTALALPADGKIT<br>AIDRDKEAYEIGLPYIRKAGVDHKI<br>NFIQSDAFSVLNDLIADSQEGGTFD<br>FAFVDAKKDDFMKYHELVLKLVK<br>VGGVIGYDDTLWFGAVALSETDD<br>MGDHLKLWRDQLREFNSFLGKDP<br>RVECCLLSVGGGLTLCRRLY*
EeOMT5	ATGGAGCGAGGCCGGGACAAGGGCGAGATCCTG<br>GCAAGCAAAGCTCTCTCGAAGTACATATTGGAGA<br>CGAATGCATATCCAAGAGAGCACGAGCAGCTAAA<br>AGAACTCAGGGAGGCCACGGTCCAGAAGTACCA<br>AATCCGGAGTATAATGAACGTGCCGGCTGATGAG<br>GGGCAGCTAATCTCCATGATGTTGAAACTCATGAA<br>TGCAAAGAAAACAATCGAGATCGGAGTCTTCACG<br>GGCTATTCTCTTCTCACCACCGCACTTGCACTTCC<br>GGCCGACGGCAAGATAATAGCGATAGACCCGGAT<br>AAGGAGGCCTATGAAATTGGCCTGCCATATATCCG<br>AAAAGCCGGAGTCGATCATAAGATCAACTTCATC<br>CAATCGGATGCCTTCTTGGTTCTGAACGACCTTAT<br>CGCGGATAGCCAAGAAGAGGGGACCTTTGATTTT<br>GCTTTCGTGGATGCCAAGAAGGACGACTACATGA<br>AATACCACGAGCTGGTGCTCAAGCTGGTGAAGGT<br>CGGAGGAGTGATCGGGTACGACGACACCCTGTGG<br>TTTGGTGCGGTCGCGCTCTCCGAAACCGATGAGA<br>TGGGCGACCAACTGAAGCTCTGGAGAGACCAAC<br>TCAGGGAGTTCAACAGCTTCGTGGCGAAGGATCC<br>GCGTGTCGAGTGCTGTCTTCTTCCTGTCGGAGGTG<br>GCCTCACGCTCTGTAGGCGCCTCTTTTAG	MERGRDKGEILASKALSKYILETNA<br>YPREHEQLKELREATVQKYQIRSIM<br>NVPADEGQLISMMLKLMNAKKTI<br>EIGVFTGYSLLTTALALPADGKIIAI<br>DPDKEAYEIGLPYIRKAGVDHKINF<br>IQSDAFLVLNDLIADSQEEGTFDFA<br>FVDAKKDDYMKYHELVLKLVKVG<br>GVIGYDDTLWFGAVALSETDEMGD<br>QLKLWRDQLREFNSFVAKDPRVEC<br>CLLPVGGGLTLCRRLF*

EeCHI1	ATGGCGCCGCCCCCTTCCGTCGCCGTCGGCGAGG<br>TCCAGGTCGAGTCCGTCAAGTTCCCGCCGTCCGT<br>CAAGCCGCCTGGCTCCGCCAAGACCCTCTTCCTC<br>GGCGGCGCAGGGGCGAGAGGCCTGGAGATCCAG<br>GGGAAGTTCATCAAGGTCACGGCGATCGGGGCCT<br>ACTTGGAGGACGCGGCGCTGCCGTCGCTCGCCGC<br>CAAGTGGAGCGGCAAGAGCGCGGAGGAGCTGGC<br>CGACTCCGTCGAGTTCTTCCGAGACGTCGTCACA<br>GGTCCATACGAGAAATTGTCCTGGGTGACCATGAT<br>ATTACCATTGACGGGACCTCAATACGCAGAGAAG<br>GTCACGGAGTACTGCGTCAAAATTTGGCAATCTGT<br>TGGAACCTACACTGATGCTGAGGCTGTTGCGGTTG<br>AGAAATTCCGCGAGGCTTTCAAGGACCAAACCTT<br>CCCACCCGGCGCCTCGATTCTATTCACACTATCGC<br>CCAACGGCTCATTGACGGTTGCATTCTCTGAAGAT<br>GGATCCGTGCCTGAAGCCTCGAACACAGTGATCG<br>AGAACAGACAACTCGCGGAGACGCTATTCGAGTC<br>GACGATTGGCAAGCACGGCGTATCTCCTGAAGCT<br>CCGACGAACCTGGCCTCGAGGATCAGTGAACTCC<br>TGAAGGACTGCGAGAAGAAGGCGGATGAAGTCG<br>ACAAGAATGCAGCCCCGAAGCCGGCGGCCAAAG<br>TCGACGTCGCGTAG	MAPPPSVAVGEVQVESVKFPPSVKP<br>PGSAKTLFLGGAGARGLEIQGKFIK<br>VTAIGAYLEDAALPSLAAKWSGKS<br>AEELADSVEFFRDVVTGPYEKLSW<br>VTMILPLTGPQYAEKVTEYCVKIW<br>QSVGTYTDAEAVAVEKFREAFKDQ<br>TFPPGASILFTLSPNGSLTVAFSEDG<br>SVPEASNTVIENRQLAETLFESTIGK<br>HGVSPEAPTNLASRISELLKDCEKK<br>ADEVDKNAAPKPAAKVDVA*

Table S4. Summary of predicted proteins structurally similar to EnOMT1 identified using the AlphaFold Database (AFDB). Gene IDs correspond to loci from the *E. grandis* reference genome. Average pLDDT represents the mean confidence score (0-100%) across all residues, with higher values indicating greater confidence in the predicted protein structure. Proteins with an average pLDDT >90% were considered high-confidence structural predictions. The top candidate OMTs, EUGRSUZ_K00951 and EUGRSUZ_F03794, which were also detected in RNA-seq datasets, were cloned and heterologously expressed. No methylation activity was detected for EUGRSUZ_K00951 against the tested panel of flavonoid substrates; therefore, this candidate was excluded from subsequent experiments.

AFDB accession	Gene ID (*E. grandis*)	Amino acid length	Average pLDDT	Presence in leaf RNAseq
AF-A0A058ZZ61-F1	EUGRSUZ_K00951	360	93.8	Y
AF-A0A059BXD9-F1	EUGRSUZ_F03794*	364	93.0	Y
AF-A0A059DG66-F1	EUGRSUZ_A01846	361	92.9	N
AF-A0A059A0D9-F1	EUGRSUZ_K00950	349	92.9	N
AF-A0A059B5G4-F1	EUGRSUZ_H03926	364	92.4	N
AF-A0A059DH23-F1	EUGRSUZ_A01796	361	92.1	N
AF-A0A058ZZ64-F1	EUGRSUZ_K00957	360	92.0	N
AF-A0A059A1C7-F1	EUGRSUZ_K00955	359	91.6	N
AF-A0A059C8F3-F1	EUGRSUZ_E03211	359	90.6	N
AF-A0A059C9A4-F1	EUGRSUZ_E03212	359	89.8	N
AF-A0A059DGJ1-F1	EUGRSUZ_A01797	370	89.1	N
AF-A0A059A0E4-F1	EUGRSUZ_K00956	361	88.8	N
AF-A0A059C6Q1-F1	EUGRSUZ_E02637	332	88.6	N
AF-A0A059B4J0-F1	EUGRSUZ_H03922	347	79.0	N

* Putative homolog of EeOMT2 (EUGRSUZ_F03794) in *E. grandis*.

Table S5. Summary of RNA-seq read mapping and feature assignment statistics. For each sample, the total number of sequenced paired-end reads and the proportion (%) of read pairs successfully aligned to the reference genome are shown.

Sample	Total reads	% of paired reads mapped to the genome	% of paired reads mapped to one feature	% of paired reads mapped to the genome but not to any feature
E. eugenioides	54,637,539	62.1	54.4	7.2
E. stenostoma	24,480,586	67.8	60.2	7.0

Table S6. List of oligonucleotides used for cloning and quantitative reverse transcription PCR (qRT-PCR).

Oligo ID	Sequence 5' to 3'	Description
EeOMT5_F	AAGCAGAATTCATGGAGCGAGGCCGGG	Cloning
EeOMT5_R	AAGCAGCGGCCGCCTAAAAGAGGCGCCTACAGAGC	
EeOMT3_F	AAGCAGAATTCATGGAGCGAGGCTGGGA	
EeOMT3_R	AAGCAGCGGCCGCCTAATAGAGGCGCCTACAAAGC	
EeOMT4_F	AAGCAGAATTCATGGAGCAAGGCTGGGAC	
EeOMT4_R	AAGCAGCGGCCGCCTAATAGAGGCGCCTACAAAGC	
EeOMT1_F	AAGCAGGTACCATGGCACCGCTCGATGAAAC	
EeOMT1_R	AAGCAGCGGCCGCTCAAGCTGGGAAGGCTTCAAT	
EeOMT2_F	AAGCAGGATCCATGGCCGACAACCAAGAAC	
EeOMT2_R	AAGCAGCGGCCGCTTAATGACCTGCCTTTTTGGGG	
EeCHI1_F	GATCCGAATTCATGGCGCCGCCCCC	
EeCHI1_R	AAGCAGCGGCCGCCTACGCGACGTCGACTTTGG	
pUbi_F	GGCAAAACCATCACCCTTGAG	
T7ter_R	AAACCCCTCAAGACCCGTTT	
A-tub_qF	CCAGTGAACAAATGCCCTCT	qRT-PCR
A-tub_qR	TGATCAGCAACAACACAGCA	
OMT1_q3F	GCATGGCGTGCTTGAATAAG	
OMT1_q3R	GTGTGGGTACAACCTGACTATC	
OMT2_q2F	GCCAATGGGAAGGTGATCGT	
OMT2_q2R	CGCAGCACTTGACTTCACAG	

Table S7. Relative methylation activity of five recombinant *E. eugenioides* OMTs toward flavonoid substrates. Activities are expressed as percentages and were normalised separately for each enzyme to the substrate showing the highest activity. Data are presented as means with 1 standard error of the mean in parentheses. Abbreviations: MT, methyltransferase; nd, no product detected for the listed methylation pattern; PCH, pinocembrin chalcone; PIN, pinocembrin; NAR, naringenin; ERI, eriodictyol; TAX, taxifolin; CHR, chrysin; API, apigenin; LUT, luteolin; QUE, quercetin.

	QUE	nd	nd	nd	nd	38.59 (12.53)	nd	9.51 (2.29)	31.48 (11.98)	11.82 (9.55)	nd

Candidate MTs	Flavonoid substrates	*O*-methylation activity (%) at different flavonoid positions									
		**7**	**7, 4′**	**7, 3′**	**7, 3′ 4′**	**3′**	**4′**	**3′,4′**	**3, 3′**	**3, 3′,4′**	**5**
*Ee*OMT1	PCH	45.98 (0.22)	nd	nd	nd	nd	nd	nd	nd	nd	nd
	PIN	100 (0.00)	nd	nd	nd	nd	nd	nd	nd	nd	nd
	NAR	81.46 (12.53)	0.48 (0.22)	nd	nd	nd	nd	nd	nd	nd	nd
	ERI	88.72 (0.54)	2.15 (0.12)	2.74 (0.18)	3.48 (0.44)	nd	nd	nd	nd	nd	nd
	TAX	35.75 (2.72)	nd	nd	nd	nd	nd	nd	nd	nd	nd
	CHR	43.87 (8.45)	nd	nd	nd	nd	nd	nd	nd	nd	nd
	API	82.78 (0.29)	7.61 (2.34)	nd	nd	nd	nd	nd	nd	nd	nd
	LUT	55.91 (2.45)	nd	6.33 (0.29)	8.74 (3.70)	nd	nd	nd	nd	nd	nd
	QUE	76.49 (5.31)	nd	4.48 (0.03)	5.78 (1.16)	nd	nd	nd	nd	nd	nd
*Ee*OMT2	PCH	nd	nd	nd	nd	nd	nd	nd	nd	nd	27.12 (0.37)
	PIN	1.12 (0.02)	nd	nd	nd	nd	nd	nd	nd	nd	4.49 (0.13)
	NAR	nd	nd	nd	nd	nd	nd	nd	nd	nd	nd
	ERI	nd	nd	nd	nd	100 (0.00)	nd	nd	nd	nd	nd
	TAX	nd	nd	nd	nd	61.89 (2.35)	nd	nd	nd	nd	nd
	CHR	nd	nd	nd	nd	nd	nd	nd	nd	nd	nd
	API	nd	nd	nd	nd	nd	nd	nd	nd	nd	nd
	LUT	nd	nd	nd	nd	87.80 (0.83)	nd	nd	nd	nd	nd
	QUE	nd	nd	nd	nd	91.63 (0.74)	nd	nd	nd	nd	nd
*Ee*OMT3	PCH	nd	nd	nd	nd	nd	nd	nd	nd	nd	nd
	PIN	nd	nd	nd	nd	nd	nd	nd	nd	nd	nd
	NAR	nd	nd	nd	nd	nd	nd	nd	nd	nd	nd
	ERI	nd	nd	nd	nd	15.27 (0.41)	nd	nd	nd	nd	nd
	TAX	nd	nd	nd	nd	14.48 (0.27)	nd	nd	nd	nd	nd
	CHR	nd	nd	nd	nd	nd	nd	nd	nd	nd	nd
	API	nd	nd	nd	nd	nd	nd	nd	nd	nd	nd
	LUT	nd	nd	nd	nd	100 (0.00)	nd	nd	nd	nd	nd
	QUE	nd	nd	nd	nd	87.41 (4.32)	nd	nd	nd	nd	nd
*Ee*OMT4	PCH	nd	nd	nd	nd	nd	nd	nd	nd	nd	nd
	PIN	nd	nd	nd	nd	nd	nd	nd	nd	nd	nd
	NAR	nd	nd	nd	nd	nd	nd	nd	nd	nd	nd
	ERI	nd	nd	nd	nd	27.51 (0.72)	3.23 (0.06)	nd	nd	nd	nd
	TAX	nd	nd	nd	nd	18.87 (0.40)	nd	nd	nd	nd	nd
	CHR	nd	nd	nd	nd	nd	nd	nd	nd	nd	nd
	API	nd	nd	nd	nd	nd	nd	nd	nd	nd	nd
	LUT	nd	nd	nd	nd	94.03 (0.41)	nd	5.97 (0.41)	nd	nd	nd
	QUE	nd	nd	nd	nd	57.49 (7.77)	nd	nd	25.72 (1.34)	3.36 (0.39)	nd
*Ee*OMT5	PCH	nd	nd	nd	nd	nd	nd	nd	nd	nd	nd
	PIN	nd	nd	nd	nd	nd	nd	nd	nd	nd	nd
	NAR	nd	nd	nd	nd	nd	nd	nd	nd	nd	nd
	ERI	nd	nd	nd	nd	78.21 (2.226)	1.71 (0.38)	12.16 (6.04)	nd	nd	nd
	TAX	nd	nd	nd	nd	75.22 (4.89)	nd	nd	nd	nd	nd
	CHR	nd	nd	nd	nd	nd	nd	nd	nd	nd	nd
	API	nd	nd	nd	nd	nd	nd	nd	nd	nd	nd
	LUT	nd	nd	nd	nd	74.93 (9.06)	nd	22.19 (6.19)	nd	nd	nd
	QUE	nd	nd	nd	nd	38.59 (12.53)	nd	9.51 (2.29)	31.48 (11.98)	11.82 (9.55)	nd

Table S8. Enzyme kinetics parameters for *Ee*OMT2 activity on pinocembrin chalcone. Ten different substrate concentrations were used ranging from 10 to 588 μM. Kinetics parameters and associated statistics were calculated using SigmaPlot version 16 Enzyme kinetics module: Single substrate, Michaelis-Menten equation. Shapiro-Wilk was used for Normality Tests. SE = standard error.

<table>
<tr><td rowspan="2">Duplicate number</td><td rowspan="2">R^2</td><td colspan="4">Michaelis constant</td><td colspan="2">ANOVA</td><td colspan="2">Normality</td></tr>
<tr><td>K_M</td><td>SE</td><td>t</td><td>P</td><td>F</td><td>P</td><td>W</td><td>P</td></tr>
<tr><td>1</td><td>0.92</td><td>210.0</td><td>84.3</td><td>2.5</td><td>=0.04</td><td>202.0</td><td><0.0001</td><td>0.97</td><td>0.88 (passed)</td></tr>
<tr><td>2</td><td>0.91</td><td>219.5</td><td>100.0</td><td>2.2</td><td>=0.06</td><td>160.1</td><td><0.0001</td><td>0.95</td><td>0.64 (passed)</td></tr>
</table>

Kinetics parameters					
V_{max} (M min^{-1})	SE	k_{cat} (min^{-1})	SE	k_{cat} / K_M (μM^{-1} min^{-1})	SE
1.3×10^{-6}	2×10^{-7}	0.049	0.009	2.3×10^{-4}	1.1×10^{-4}

Table S9. Comparison of literature ^{1}H and ^{13}C NMR data with the observed experimental NMR data for compounds 1 (methanol-d_4) and 2 ($CDCl_3$).

	**This work**		**Literature**	
Compound 1	methanol-d_4		Ref.[1]: DMSO-d_6	
Position	500 MHz δ_H (multiplicity, J Hz) ppm	125 MHz δ_C ppm	200 MHz δ_H (multiplicity, J Hz) ppm	20 MHz δ_C ppm
1	–	–	–	–
2	5.43 (dd, 12.5, 3.2)	79.8	6.49 (q, 12, 3.3)	77.8
3	2.75 (dd, 16.7, 3.2),<br>2.95 (dd, 16.7, 12.5)	46.3	2.67 (q, 16.4, 3.3),<br>2.92 (q, 16.4, 12)	44.9
4	–	192.4	–	187.9
5	–	161.7	–	159.7
6	6.13 (s)	93.5	6.17 (s)	92.9
7	–	165.0	–	161.4
8	–	105.7	–	104.7
9	–	163.7	–	162.2
10	–	105.7	–	103.4
Me-8	2.01 (s)	8.0	1.93 (s)	8.1
Me-5	3.80 (s)	55.9	3.70 (s)	55.5
1′		140.9	–	139.7
2′,6′	7.50-7.52 (m)	127.1		126.2
3′,5′	7.40-7.43 (m)	129.7	7.3-7.5 (m)	128.7
4′	7.34-7.37 (m)	129.4		128.2
Compound 2	*CDCl*$_3$		Ref.[2]: DMSO-d_6	
Position	500 MHz δ_H (multiplicity, J Hz) ppm	125 MHz δ_C ppm	500 MHz δ_H (multiplicity, J Hz) ppm	125 MHz δ_C ppm
1				
2	5.42 (dd, 13.1, 3.0)	79.4	5.5 (dd, 12.9, 3.0)	79.1
3	2.82 (dd, 17.1, 3.0),<br>3.09 (17.1, 13.1)	43.5	3.1 (dd, 17.1, 3.0),<br>2.8 (dd, 17.1, 3.0)	42.8
4		195.8		
5		160.4		161.5
6	6.10 (s)	90.8	6.2 (s)	90.6
7		165.8		165.9
8		106.1		105
9		161.2		159.9
10		102.8		102.4
Me-8	2.01 (s)	6.9	2.0 (s)	5.9
Me-5	3.84 (s)	55.8	3.9 (s)	55.1
1′		138.5		139.1
2′,6′		126.2	7.4 (m)	126.1
3′,5′	7.37-7.48 (m)	128.9	7.5 (dd, 7.8, 3.4)	128.4
4′		128.9	7.4 (d, 7.4)	125.9

References

1. Markham, K. R.; Wollenweber, E.; Schilling, G., Structure revision for two *C*-methyl flavanones from *Pityrogramma pallida*. *Journal of Plant Physiology* **1987,** 131, (1), 45-48.
2. Massaro, C. F.; Katouli, M.; Grkovic, T.; Vu, H.; Quinn, R. J.; Heard, T. A.; Carvalho, C.; Manley-Harris, M.; Wallace, H. M.; Brooks, P., Anti-staphylococcal activity of *C*-methyl flavanones from propolis of Australian stingless bees (*Tetragonula carbonaria*) and fruit resins of *Corymbia torelliana* (Myrtaceae). *Fitoterapia* **2014,** 95, 247-57.
